# A review of anti-surge control systems of compressors and advanced fault-tolerant control techniques for integration perspective

**DOI:** 10.1016/j.heliyon.2023.e19557

**Published:** 2023-08-28

**Authors:** Turki Alsuwian, Arslan Ahmed Amin, Muhammad Sajid Iqbal, Muhammad Taimoor Maqsood

**Affiliations:** a Department of Electrical Engineering, College of Engineering, Najran University, Najran 11001, Saudi Arabia; b Department of Electrical Engineering, FAST National University of Computer and Emerging Sciences, Chiniot Faisalabad Campus, Chiniot 35400, Punjab, Pakistan

## Abstract

In this review paper, the anti-surge control (ASC) of the compressors and fault-tolerant control (FTC) systems are described from the perspective of integration for reliability enhancement against faults in the system components. It explains the phenomenon of a surge in the compressors, surge precursors, and the potential damage caused by this surge. The explanation of surge avoidance methods in compressors incorporated with modern surge control systems is described along with their applications. The sizing of the system components and valves, particularly methodologies for appropriately estimating acceptable upstream pipe sizes, are elaborated. The existing surge protection techniques for compressors are analyzed to highlight the advantages and disadvantages and from a future perspective, new approaches for detecting system changes and surges, are included. In the end, the concept of fault tolerance and its advanced applications concerning the anti-surge control for compressors are explained. This study contributes to the young researchers in the field of anti-surge control systems for compressors with the integration of fault tolerance to increase the reliability of the system.

INDEX TERMS Compressor Control System; Anti-Surge Control; Fault Tolerance; Fault-Tolerant Anti-Surge Control.

## 1 Introduction

### 1.1 Compressors

Compressors are extensively utilized in the oil and gas industry where the gas is needed to be transported for longer distances by enhancing the pressure [1]. The centrifugal gas compressor working principle can be described by some important operational constraints: flow, speed, and head. A maximum head of centrifugal compressors can be achieved at some desired speed. In that region, there exists an equivalent flow, known as the limit of stability. The operation of the compressor is steady if the head is lesser i.e. showing low friction with the compressor, and the flow is higher than the low limit. In other words, the stability of the system is not compromised if the head reductions cause a flow increment. When the maximum load capacity of a compressor is achieved and the flow reaches a minimum value, a surge occurs. Considering the dynamic nature of the compressors, a surge of the system may take place at slightly greater than the maximum head capacity with low flow rates, hence, this is a specific problem that usually occurs in low-frequency pulse systems [2]. The flow is reversed when the compressor is unable to meet the head forced by the conditions of discharge and suction, as implemented by the system of compression.

### 1.2 Surge phenomenon in compressors

Surge is a condition in compressors in which the flow is reversed, and the compressor speed reaches the speed of sound. The whole process is repeated, and this cyclic phenomenon causes a lot of damage to the compressor, and human injury is also expected. As it is a natural phenomenon, avoidance needs to be designed to protect the compressor from a surge condition. As the minimum value of flow is reached, the surge occurs. There are different operating conditions where a surge may occur considering the uncertainty in volumetric flow, suction pressure, the difference in discharge and header pressure, the startup of the compressor, the emergency shutdown of the compressor, and other parameters [1]. A surge can cause oscillations in the system and not only the compressor is affected but the nearby piping can also be damaged which may be attached to some other critical systems as well and a hazardous condition may occur, reducing the overall efficiency of the installed system [3].

When the surge limit of a compressor is reached, components like impellers and diffusers can move to stall conditions. The separation of gas from the surface of the flow can cause a stall. Whenever the operating point of a compressor is varied, the aerodynamic components' incident angle is also varied. Like in an airfoil, an increment in the angle of the incident ultimately causes a stall. The stall in turbomachines is frequently shown as a condition in rotation, when confined areas of separate flow, at lower speeds compared to impeller rotational speed, travel with the diffuser [4]. The system's stability is compromised due to the surge.

Stability in the process can be disturbed while trying to increase the series resistance with the compressor such that the compressor head reaches its peak value. The flow rate will decline in this condition and therefore, the head capacity of the compressor will tend to decline. As the capacity of the head declines, the flow declines more. As soon as the compressor is unable to reach the external head, the flow reversal occurs. Once the compression system stability limit is exceeded, the surge occurs. Not only does this hamper the achievement of the process objectives, but the resulting radial and axial rotor movements can damage the compressor, sometimes relentlessly. Therefore, flow is maintained at a rate such that it does not decrease more than the peak head flow to avoid the surge.

### 1.3 Surge control applications

To better comprehend the compressor surge control system working conditions, typical applications necessitating the use of surge control systems are outlined below.

**Pipeline Compression:** Centrifugal compressor surge systems are commonly used in pipeline compression applications. The pressure ratio across the compressor in these setups varies depending on the pipeline. Surges will occur in pipeline applications owing to flow decreases managed by the pipeline. These will happen over the whole range of the compressor's pressure ratio [5]. The occurrence of a surge will cause changes in gas composition and operating temperature so these uncertainties should be included in the surge margin.

**Re-Injection:** The compressor is used in re-injection applications to inject gas back into a potential producing field. These applications necessitate a variable discharge pressure dependent on the gas field pressure requirements, which fluctuate over time. As there are many sources of gas, the composition of the gas might vary greatly, necessitating a depiction of the surge line in reduced head and flow (composition independent) form. Surge control devices may be used to limit the effects of liquid slugs of gas that would create flow interruptions. Because the discharge gas pressure can be quite high (more than 3000 psi) and often involves many bodies, the pressure ratio is much higher in this sort of application [5]. Extreme temperature swings are uncommon in this sort of application, but they might occur on a seasonal basis. At times, rapid fluctuations in suction flow might occur owing to needed valve manipulations and process disruptions.

**Storage and Withdrawal:** The compressor is used to inject or remove gas from a storage field or reservoir in storage and withdrawal applications. The suction gas pressure fluctuates throughout time (initially high but declining quickly over time for withdrawal applications or initially low and increasing gradually in injection applications.) The starting pressure in the storage field is also used to calculate the pressure ratio. The fluctuations in gas composition are caused by changes in the storage gas. These applications can be particularly difficult for a surge control system since they require a quick reaction for regular process management, but a slower response when the pressure ratio declines. As a result, the recycling valve's control signals may need to be more diversified than in a normal pipeline application.

**Gas Gathering:** To prepare the gas for processing or transmission pipeline applications, gas collection facilities collect production-type gas mixes. The types of gas being treated, as well as the quantity of dehydration, separation, and filtration utilized at one specific installation, may create large fluctuations in gas composition in this application. Variations in gas mixture owing to diverse production fields will induce changes in the compressor's typical head vs. speed curves. Because the discharge gas is often delivered into a pipeline system, the pressure ratios are typically lower than in re-injection and storage applications, with discharge pressures kept below 3000 psi. In this application, the gas temperatures are consistent.

**High Pressure/Process Compression:** Depending on the application, high-pressure compressors have a wide range of pressure ratios and gas compositions; higher pressures are often in the range of 3000–5000 psi [5]. Depending on the kind of process, the pressure, head, and operational parameters of the processing plant compressor will fluctuate (LNG facilities, refineries, hydrocarbon processing, and NGL removal processes). The composition and temperature of the gas will also change depending on the process needs. Surge control systems should be designed to eliminate surges during normal operation since greater pressures increase the danger of compressor failure.

In this paper, our contribution is the unique integration of ASC of the compressors and FTC systems for reliability enhancement against faults in the system components. The paper explains the phenomenon of a surge in the compressors briefly, surge precursors, and the potential damage caused by this surge. The explanation of surge avoidance methods in compressors incorporated with modern surge control systems is described along with their applications. The design aspects regarding sizing of the system components and valves, particularly methodologies for appropriately estimating acceptable upstream pipe sizes, are elaborated. The existing surge protection techniques for compressors are analyzed to highlight the advantages and disadvantages and from a future perspective, new approaches for detecting system changes and surges, are included. This study contributes to the young researchers in the field of anti-surge control systems for compressors with the integration of fault tolerance to increase the reliability of the system.

**Fig. 1:**
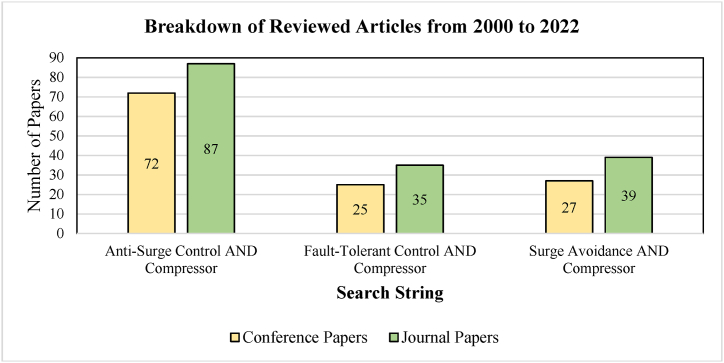
Distribution of Articles found in the Scopus database.

In order to conduct this review, we have used the Scopus database to find the relevant research paper. Different search strings were used to find the relevant research articles from the year 2000–2022. The details of the search strings applied, and the results found on the Scopus database for the above-mentioned years are shown in Table 1 and their distribution is shown in Fig. 1.

**Table 1:** Search Strings and Research Articles found in Scopus.

Search String	Conference Papers	Journal Papers	Total	Grand Total
Anti-Surge Control AND Compressor	72	87	159	**285**
Fault-Tolerant Control AND Compressor	25	35	60	
Surge Avoidance AND Compressor	27	39	66

The articles were divided into two categories against each search string conference paper and journal paper. We have found a total of 285 articles related to our review topic. Most relevant articles were then further reduced to 112 according to the following criteria.

• The redundant papers were removed from the obtained results.
• More preference was given to journal articles, there was a total of 161 journal articles, out of which 98 were used in this review based on the publication date, journal type, and relevance to the topic. Moreover, the recent article is selected from an author if there were more than 1 article from the same author.
• Ten conference papers were also included in the review. The papers from the conferences with a long sequence were preferred.
• Apart from the conference and journal articles, 3 patents are also included in the review.

Further contents of the paper are organized as: section 2 explains the surge prevention methodology with design considerations, section 3 explains the detailed ASC design aspects with a brief discussion on instrumentation selection, and section 4 explains the FTC systems with possible types, integration with the ASC systems and future research directions. The article is finally concluded with future directions.

## 2 Surge prevention methodology

By installing a control valve in the compressor as a bypass, the surge can be prevented evidently. The temperature and pressure transmitters are installed on compressor discharge and suction lines in a common compressor system, with a differential pressure transmitter for the measurement of flow through the compressor, a control valve for surge protection, and a control system algorithm.

An anti-surge system defines the operating point of the compressor with the collection of instrumentation data for flow, temperature, and pressure. The compressor surge limit is compared to the operating point of the system. The control error is defined by calculating the variance between the surge limit and the operating point. To produce a control signal for the operation of the recycle valve, a PID control algorithm is usually defined. When the recycle valve is opened, some gas proportion is headed again to the suction side from the discharge, and the increase in the head is avoided in this case. When the flow is increased above the normal operating point then the valve is made to close and the normal operation of the compressor continues [[6], [7], [8], [9], [10]].

Different approaches have been taken to avoid the surge in the compressor. The occurrence of a surge produces greater chances of the instability of the entire system, hence either the gas is exposed to the air which is dangerous as it is explosive, or a recycle valve is installed in the compressor to operate in surge conditions so that the damage is avoided [1,11].

### 2.1 Important elements of anti-surge control design

Five important elements to effectively avoid the surge as used in Anti-Surge Control (ASC) design are explained here [12].

i . An accurate surge limit model: It should provide the surge limit within the appropriate limits of the characteristics and conditions of the gas.
ii . Accurate control algorithm: The surge must be avoided with the process variations.
iii . Proper instrumentation: Accuracy, range, and speed must be the priority when selecting the instrumentation.
iv . Correctly selected recycle valve for the compressor: The valves must be suitable for the compressor. They must be able to make rapid and large changes in capacity.
v . Properly selected recycle valve for system volumes: Surge limit should not be attained in the case of a shutdown; hence the speed of the valve should be fast and large. The response of the system highly depends upon the piping of the system. It should be kept in mind and analyzed. A single-valve anti-surge system is avoided in larger volumes of pipes.

In early manufacturing, the surge controls were operated pneumatically. The parameter to watch, to control the surge, was a differential pressure (DP) through the flow measuring device by the source of a beam controlling the signal to the recycle valve and DP across the compressor. The advanced performance and the high-stress-bearing compressors needed a high-quality surge protection system. As the technology of microprocessors arrived, the intensity of the arithmetic calculations increased, the modeling was based on polynomials for the surge limit, and unbalanced control systems were introduced. Surge control of the modern area focuses on maximizing the compressor operating zone [[12], [13], [14], [15], [16]]. The latest techniques of the ASC system can be reviewed in Refs. [[17], [18], [19]].

### 2.2 Anti-surge control system model

**Fig. 2:**
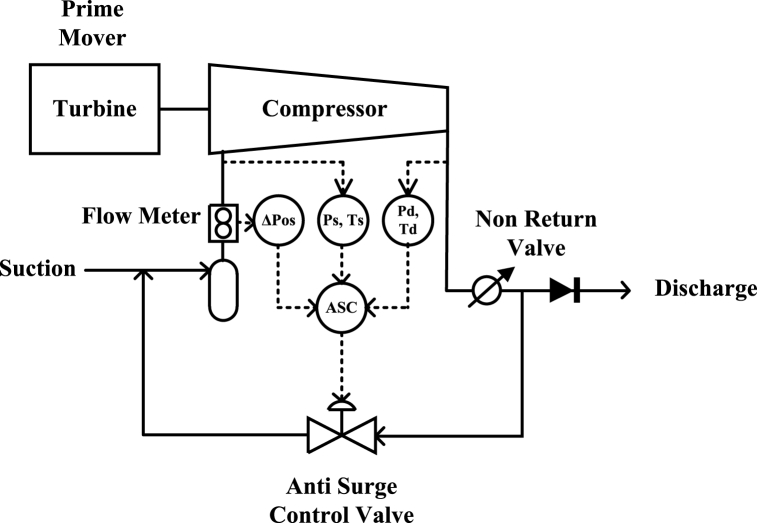
Compressor Control System Model [1].

ASC system is used to prevent the event of a surge to occur in the compressors. These ASC systems should be quick enough to respond with a very minute effect on the process. The conditions of the surge occurrence must be known to avoid it. The accuracy in it would lead to a better operating zone for a compressor. The parameters, by which the compressor operating zone is defined, are the speed, head, and flow. The surge and the operating zone of the compressor can be derived in a mathematical relation using the above parameters. A compressor's complete layout is shown in Fig. 2.

**Fig. 3:**
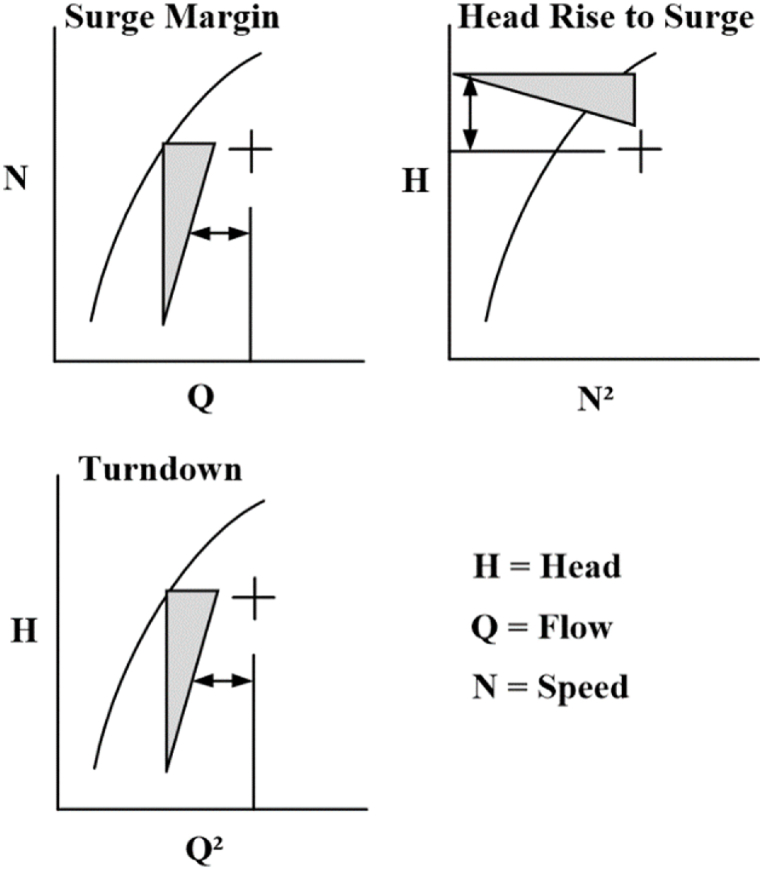
Various References for surge limit determination [6].

Various ASC models are shown in Fig. 3 in which the first two involve speed. The variation in gas composition during the operation highly affects the compressor speed, as the Mach number of the machine will be varied [21].

The H denotes the mathematical relation of head versus flow to give the modeling source of surge limit regardless of the variation in the gas properties. The measurement of surge limit model parameters can be done in terms of the head across the flowmeter and the compressor.

The calculation for the head across the compressor and the flowmeter led to some parameters like temperature, units, compressibility, and specific gravity that can be canceled out from both equations to reduce the equation into a simpler form and the resulting equation can be termed as reduced head versus reduced flow as shown in Equation (2).

(1) HP=K.(PDPS)σ−1σ.T.SG.Z

(2) $$H_{Reduced}=\frac{{\left(\frac{P_{D}}{P_{S}}\right)}^{\sigma }-1}{\sigma }$$

Different relations involve in terms of defining the operating zone of the compressor. The ratio of pressures for the compressor “*R*_*c*_” can be seen below [1]:

(3) $$R_{C}=\frac{P_{d}}{P_{s}}$$

(4) $$\sigma =\log\left\{\left(\frac{T_{d}}{T_{s}}-\frac{P_{d}}{P_{s}}\right)\right\}$$

(5) $$qr^{2}=\frac{\Delta P_{os}}{P_{s}}$$

Where *P*_*d*_ is the discharge pressure of the compressor, *P*_*s*_ is the suction pressure, *σ* is the polytrophic head exponent, *T*_*d*_ is the discharge temperature, *T*_*s*_ is the suction temperature, *qr*^*2*^ is the reduced flow and *ΔPos* is the DP across the flowmeter. Hence the *P*_*d*,_ *P*_*s*,_ *T*_*d*,_ *T*_*s*,_ and *ΔPos* are the five sensor values that are used to define the algorithm to control the actuator.

Different authors have proposed different surge models for the compressor and implemented these in various environments; some of them have been discussed here. In Ref. [21], the compressor model has been developed for automotive engines and their model is predictive of the change in parameters downstream of the compressor, and its validation is tested upon the low and high frequencies. In Ref. [3], a dynamic model of the compressor has been implemented for different speeds, the surge phenomenon is tested by tuning the ducts and the compressor losses have been tested. In Ref. [22], the thermodynamics and fluid mechanics of the compressor are examined for the surge process, and real-time simulation-based testing is performed which proved that their model is predictive of the dynamic parameters of the compressor for the surge.

**Fig. 4:**
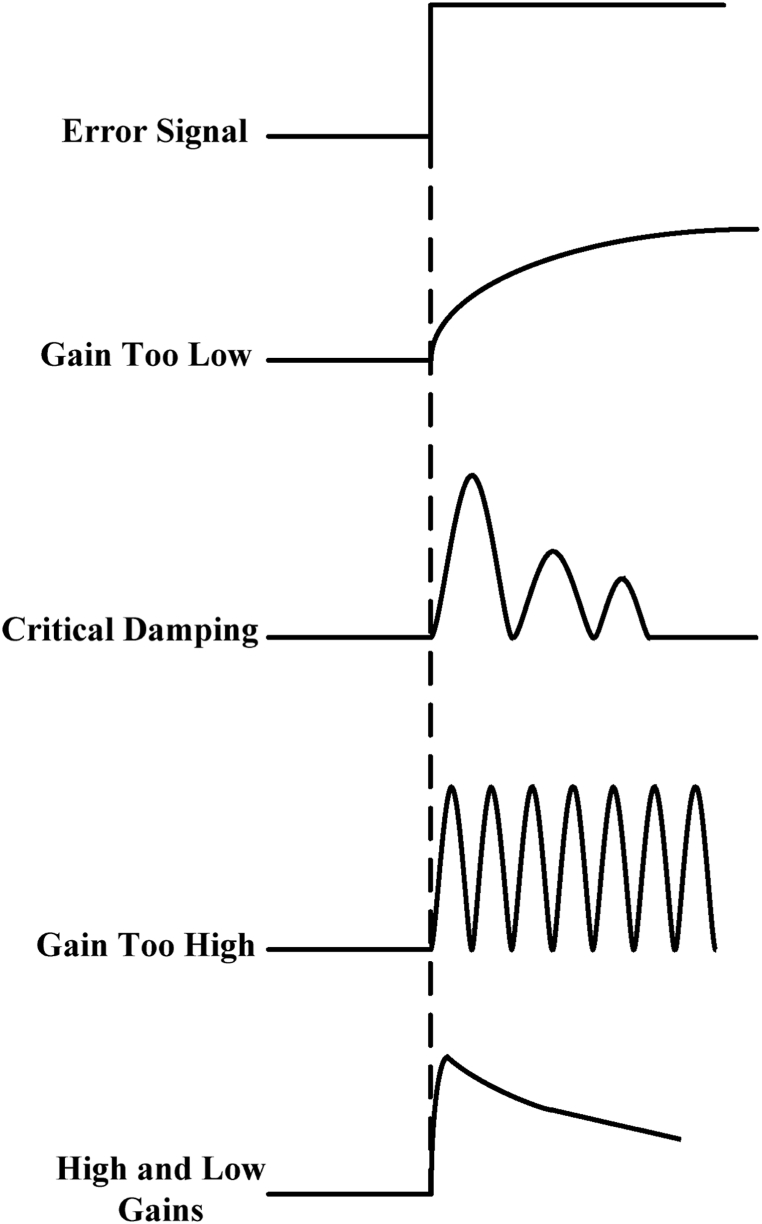
Control System Responses To Error Signal [6].

Control response is generated due to a rapid change in the system. Tuning done in various methods for the controls has been shown in Fig. 4.

A slow response is generated with low gains. An aggressive response is generated by the critically damped response with a quick final steady state. Oscillations in the system tend to happen when the gains are set very high [23].

As the surge avoidance system is most of the time idle and waiting for the surge to occur, therefore, without showing any deviation it must take the action aggressively in no time for the compressor protection. This might require very high gains within the stability zone. The gains are reduced to evade the instability outside of the system. As soon as the surge is prevented by the anti-surge system, the process should be resumed by the control system at an optimum rate to reduce the upsets chances.

## 3 Anti-surge control system design techniques

### 3.1 Control system architectures

In [23], the ASC model is implemented and the surge is controlled using advanced torque control with variable frequency drives. In Ref. [24], the ASC model is designed, and discharge pressure regulation is done such that surge is avoided during the different changes and noises in the pipeline using the multivariable predictive control. The proposed controller can be used in coupled processes and can be implemented with the minimum possible process information. The major limitation of this system is the installation and maintenance costs and the requirement of a large number of tuning parameters. In Ref. [25], using integral control, piston-actuated ASC for the compressor is modeled and the control of the piston is the main cause to avoid the surge with its double integral control that makes it more stable where the linear-quadratic regulator algorithm is used for the optimization. The proposed controller can operate on continuous deviations and can restore the controlled variable to its original position. However, the response time for the proposed technique is very high which makes it unsuitable for time-sensitive applications. In Ref. [26], various model predictive control approaches are developed and the proposed technique proves to improve the performance of the controller in the surge avoidance scheme. In Ref. [27], different models have been discussed for ASC in compressors and the performance of control systems is increased. In Ref. [28], the active surge controller is designed and the magnetically controlled bearings are tracked. However, the system is limited and sensitive to signal-to-noise ratio. Many other models similar to these for ASC systems are explained in Refs. [11,27].

In [29], an effective surge control system is simulated for the case of emergency shutdown (ESD) and the two competing methods have been tested using effective simulation and the results are validated for the hot and cold recycle valves. In Ref. [30], the anti-surge valve is controlled using a closed-loop response given by the model and a safety margin that initiates the closed-loop response using the load variable used in the model. Even in the presence of non-linearities, the controller described in Ref. [30] produces correct results. But no comments are made about the stability of the system. In Ref. [31], the safety and reliability of the compressor surge control system are analyzed In Ref. [32], the recycle valve specifications and parameters that are to be considered while designing a compressor control system including the selection of the instrumentation are described. From the start-up to ESD, all guidelines are described from the design perspective. In Ref. [33], a model-predictive controller (MPC) is implemented for the ASC and compared with the existing PID controller. The results show that MPC is more effective than PID control by proving that their model can minimize the steady-state error and percentage overshoot. MPC's downside is its sophisticated algorithm, which takes longer than the other controllers. In Ref. [34], the compressor control system is designed by using two valves, one for slow disturbances and the other for high disturbances like ESD. Their model increased the surge margin by 10% from the surge line. In Ref. [35], the three operating zones have been simulated using variable fluids, and their behavior for the start-up, ESD, and normal operation is tested for every fluid. By comparing the results, surge avoidance is tested for each fluid. In Ref. [36], a robust controller is designed against the disturbances in the system. The controller is robust to unmodelled dynamics and therefore the compressor operating zone is expanded. In Ref. [37], the compressor's first stage outlet is fed into the next stage of the compressor, and the volumetric flow is maintained to a position where the low flow is avoided in each stage of the compressor, which avoids the surge.

In [38], the compressor model inputs and outputs are used to predict the behavior of the compressor, and a model predictive controller is designed for surge avoidance. Their model increases the efficiency of the compressor by decreasing the margin between the surge and control lines. In Ref. [39], the existing PID controller is compared with the PI Fuzzy logic controller, and the simulations exhibit that the PI Fuzzy logic controller is quicker in response and the opening of the valve is also more effective than the PID controller. However, the system's efficiency is low since it relies heavily on fault data. The knowledge and skills of humans are completely reliant on a fuzzy logic controller. Machine learning and neural networks are not recognized by these controllers. In Ref. [40], the compressor anti-surge controller and the compression model are designed and experimented with by taking the data from the industry and providing a comprehensive model for surge control. In Ref. [41], three methods have been proposed to assess the effectiveness of any compressor anti-surge control method and concluded some important parameters that are to be considered while designing a compressor anti-surge control.

**Fig. 5:**
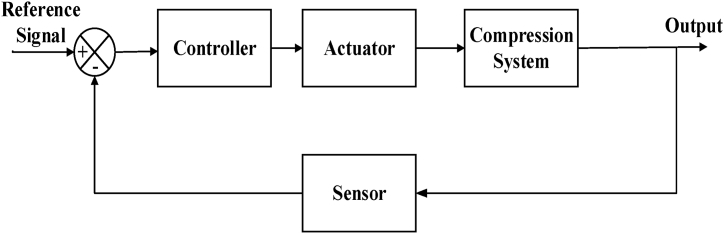
Active Anti-Surge Control Model [42].

One of the latest techniques for the active ASC scheme has been presented by Ref. [42] as shown in Fig. 5. Where the sensor reads the output and gives effective feedback to the system and the modified signal updates the controller to send the input signal to the actuator for the desired opening to avoid the surge. As far as the control approaches are concerned, different controllers are being used by many authors according to their model's requirement and efficiency, as in Ref. [26], where the model predictive control is used and is one of the most advanced controls in which the system behavior is predicted by the controller and the update occurs at every step of the process.

**Fig. 6:**
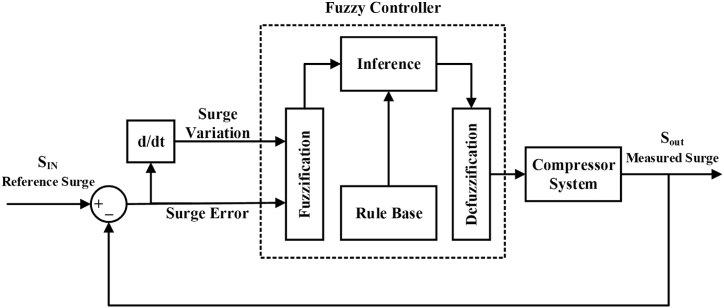
Fuzzy logic control for ASC system.

In [43], a framework is designed using fuzzy logic control in which the various faults have been tested for the surge and this method is quite useful for nonlinear systems. Moreover, the model is self-predictive such that the controller action is independent of the compressor map information and the decision is taken upon the output of the compressor. The proposed control scheme has been described in Fig. 6.

Here S_In_ is the reference surge or setpoint and S_Out_ is the measured surge of the compression system. An active fault-tolerant control system is being proposed here in which the fuzzy model is acting like an observer and the surge error is being taken by the difference between the reference surge and the surge measured by the fuzzy model. The derivative is computed and fed to the fuzzy controller again to tune the membership functions and make appropriate changes in the system to reduce the error. In this paper, the authors have tried to offer a different approach to the supervision of compression systems by using model-based fault detection and isolation, together with self-tuning of surge measurements and subsequent corrective measures that make the proposed surge protection system more robust. In Ref. [2], a model is designed for the surge in the case of an emergency shutdown of the compressor and their simulation model validates that a surge is produced when the case is achieved with its avoidance in milliseconds executed effectively.

**Fig. 7:**
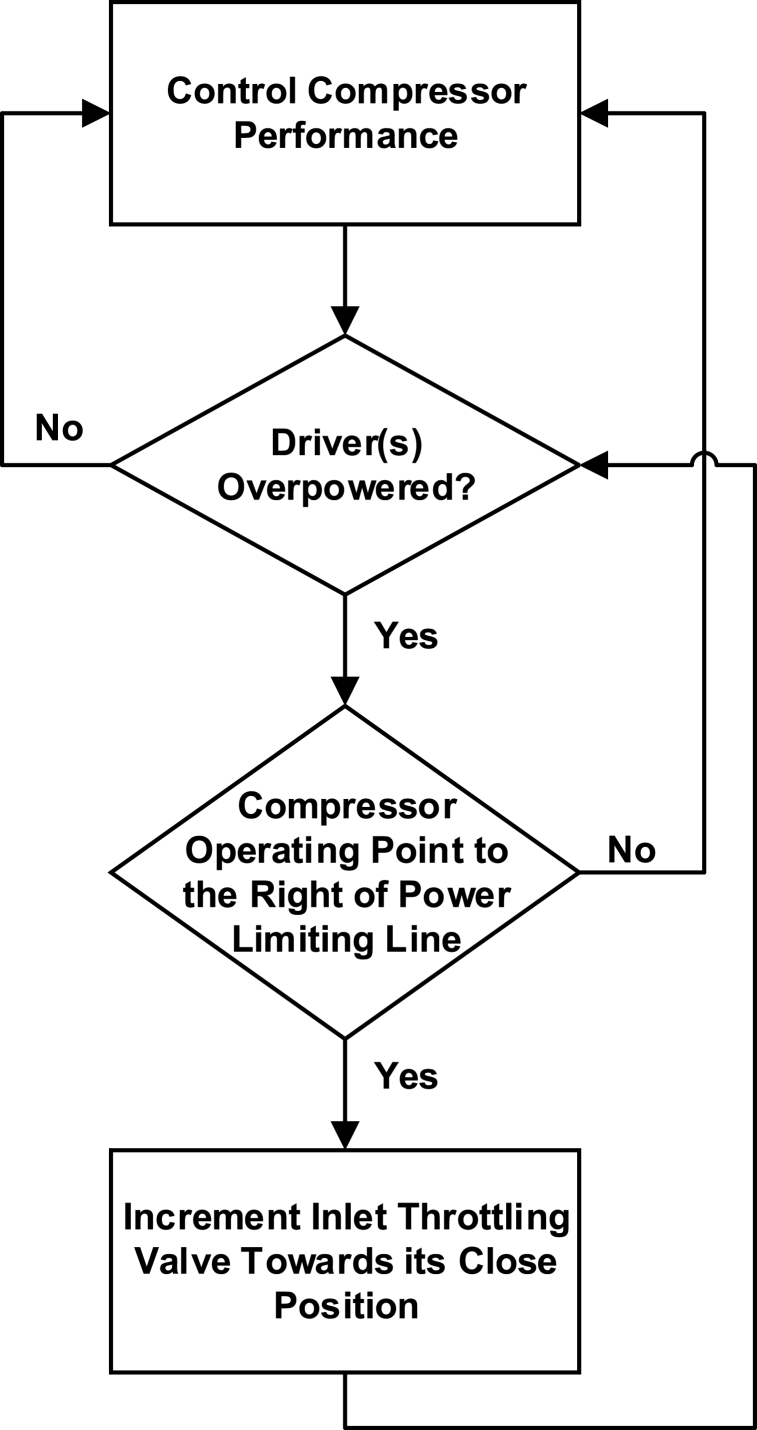
Surge Protection Mechanism [49].

The conventional algorithms are mainly based on the flow, speed, head, and pressure to form a compressor curve as discussed earlier. The variations in the operating point along the compressor curve at a speed are monitored and the surge limit is considered as the boundary of the system, below which the flow reaches the stonewall limit and the compressor experiences the surge which is then prevented by this conventional algorithm. This algorithm is old and can be reviewed in Refs. [[44], [45], [46], [47], [48]]. A mechanism and apparatus for shielding a compression device from both driver overpowering and turbo-compressor surge at the same time are presented in Ref. [49]. Normally, when overpowering is observed, the flow rate through each compressor in the turbo compressor train is decreased by closing an inlet throttling valve at the inlet of each respective compressor stage, unless the compressor operating point is too close to Surge. The inlet throttle valve is not closed in this study. To stop the surge, the average flow rate through the compressor train is decreased while enough flow is retained through the damaged phases. The surge control mechanism is shown in Fig. 7.

In [50], the authors have described different reasons for the surge occurrence. The reasons include erratic flow or stalling. The study is related to axial or radial compressors, and it is shown that surges can only be managed in axial or radial compressor installations by moving the operating point in the operating envelope to the right, away from the surge limit line. In Ref. [51], the conventional control approaches and the surge are thoroughly explained. These approaches are.

• Centrifugal Anti-surge Control based on the Constant Speed
• Centrifugal Anti-surge Control based on the Variable Speed
• Discharge Volume Flow (P and T Compensated) Anti-surge Control for Variable Speed Compressor
• Discharge Volume Flow (non-Compensated) Anti-surge Control for Variable Speed Compressor
• Flow/Delta-P Anti-surge Control

**Fig. 8:**
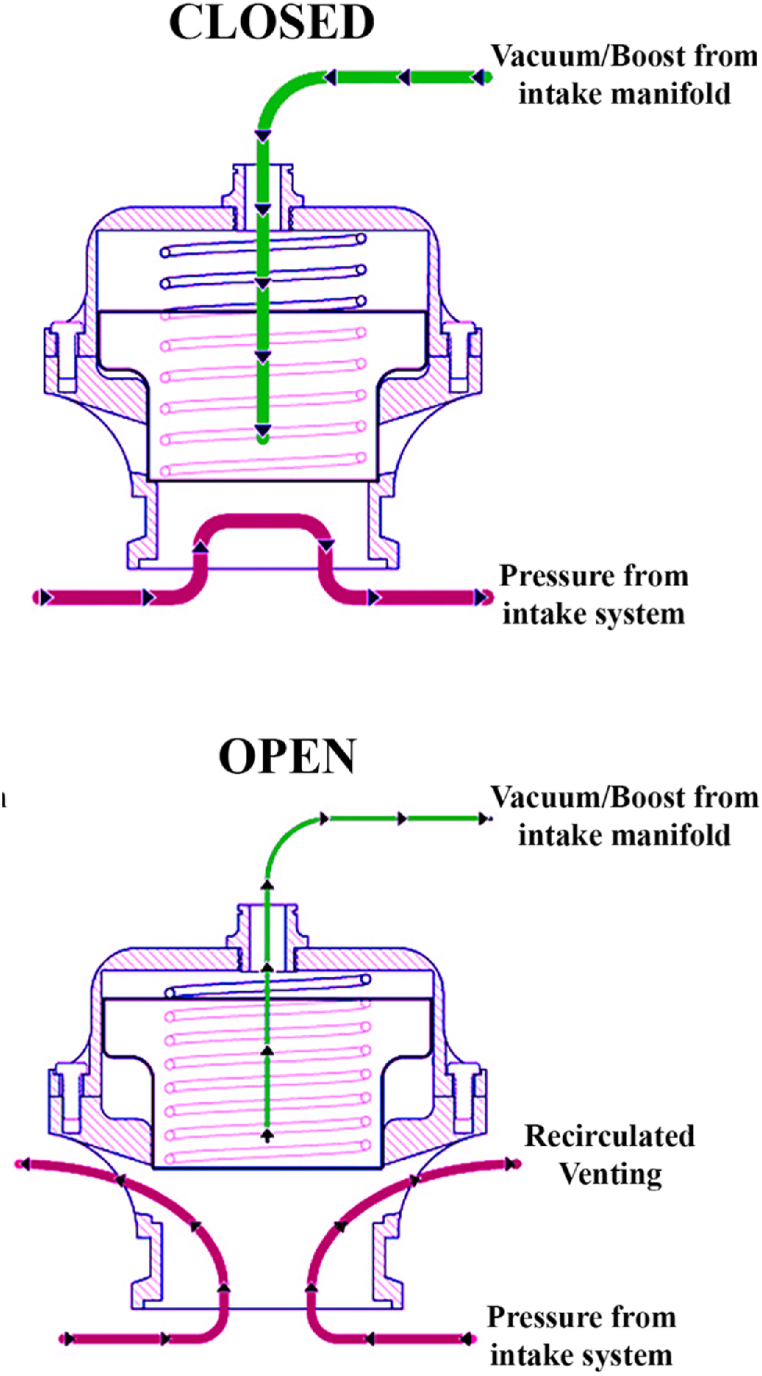
Working of a blow-off valve in closed and open situations [52].

In [52], a power-saving method is proposed while operating the ASC by proposing a blow-off valve. The working of a blow-off valve is illustrated in Fig. 8.

The advanced controller in which PI + D is used for ESD case for a quicker response as in Refs. [1,20]. The modern and advanced ASC algorithms work on other parameters and curves. The different curves other than the compressor conventional curve or map are described in Ref. [25]. The ratio between flow and speed is directly considered for the algorithm reference as in Refs. [[53], [54], [55]]. A programmable logic controller is also used but due to its complexity, it is less common as mentioned in Refs. [[56], [57], [58], [59]]. In some algorithms, the vibration of the system is also taken as the reference and the control revolves around this measurement as in Refs. [60,61]. Similarly, the temperature rise can also be considered during the surge and it can be used for the algorithm but with the pressure and speed, considerations as well as described in Refs. [[62], [63], [64]].

In [65], the authors have demonstrated surge control for a class of nonlinear systems as a novel fault detection and isolation method based on the fuzzy Takagi Sugeno model. The said method was used in the residual generation stage of the fault detection and isolation (FDI) model to produce a family of system responses from the trusted band, which includes the effects of uncertainty and is based solely on input-output results. When obtaining the input-output data set, using low-pass filtering allows for the use of a relatively simple fuzzy structure for fault detection and isolation, with no noticeable reduction in fault detection stage performance. The results suggested that the surge can be detected and isolated even if limited data for input/output is available. However, just a few simple examples were used to show the method's usefulness and usability.

**Fig. 9:**
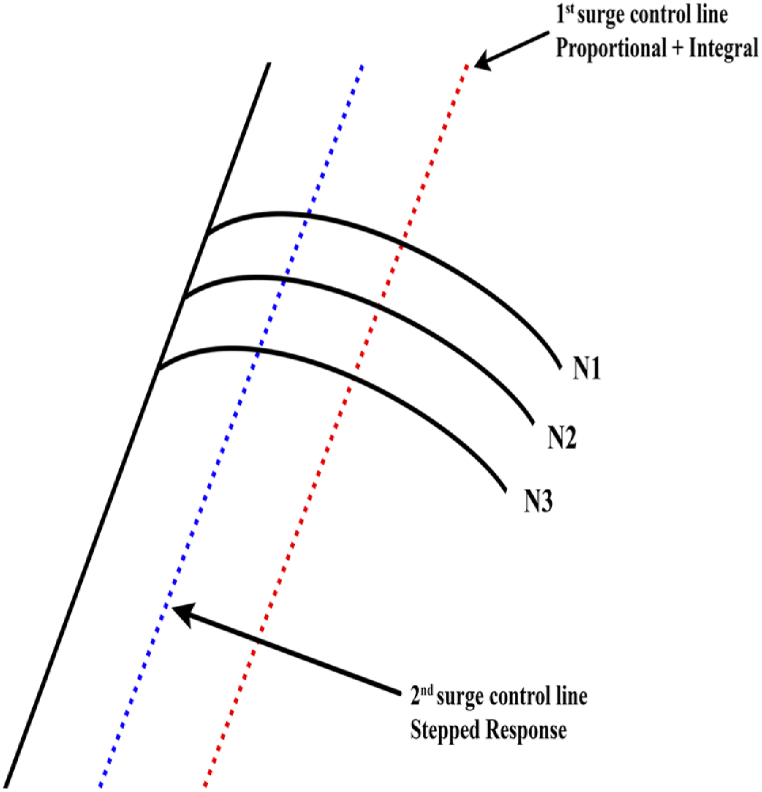
Surge Lines and PI control [67].

A variable speed drive (VSD) based soft sensing method is used in Ref. [66] to determine the leakage and calculate the pressure in compressed air systems. The idea is unique as it uses soft sensing instead of using actual sensors and produces encouraging results. The proposed idea is mainly for screw compressors but can be generalized to centrifugal compressors. The major advantage of the proposed system is that we can acquire a wide variety of speed, torque, and power outputs from VSDs, which helps us achieve better system management. VSDs serve to improve the quality of the processing system by providing a wide range of control. However, the drive system for the proposed controller will have a higher cost and harmonic currents. The authors in Ref. [67] have proposed a PI controller-based approach to protect the centrifugal compressor from a surge. The valves are operating based on the difference between the surge protection line and the compressor line. These lines are shown in Fig. 9 below.

The use of a PI controller provides an error-free steady-state response but at the same time, the stability margin decreases as PI controllers have a narrower stability range. For surge control in multi-compressor systems, a decentralized MPC controller is reported in Ref. [68]. Backstepping and MPC-based anti-surge controllers are proposed in Refs. [69,70] which are used for gas compressors and nonlinear systems, respectively. The proposed methods claim higher accuracy by keeping the tuning parameters to the minimum level. However, while designing these systems, the fluctuations of different compressor parts are not considered. The variations in different parameters like piping and upstream/downstream parts of the characteristic curve will change and hence the results of the proposed control system may change. Another MPC-based method can be found in Ref. [71] which considers the uncertainties and unmatched disturbances for the compressor. The major advantage of this approach is that it can prevent surges despite matched and mismatched disturbances in the compressor system without requiring a throttle valve value. However, the suggested approach has some significant drawbacks, including high complexity, high computational cost (which necessitates the use of expensive dedicated high-speed controllers), and high maintenance and troubleshooting costs.

In all the above-mentioned conventional and advanced techniques for compressor surge control, the surge is prevented by considering the parameters like flow, speed, head, pressure, vibration, fuzzy residuals, or gas leakage. The parameters considered for surge detection and prevention in conventional as well as advanced methods are listed in Table 2 below.

**Table 2:** Conventional and advanced methods of ASC.

Description	Parameters Considered
Conventional Methods	Flow
	Speed
	Head
	Pressure
Advanced Methods	Flow-Speed Ratio
	Programmable Logic Controller-based Control
	Vibration
	Temperature
	VFD Torque and Cool Recycle Valve
	Fuzzy Residuals
	Surge Protection Line
	Compressed air leakage

### 3.2 Instrumentation for anti-surge control system

A control system's optimal working depends upon the process instrumentation resolution, accuracy, and speed. The instrumentation must be selected at a magnitude higher than the requirement of the system in order to achieve optimal results. For example, a compressor's first-time constant is usually nearly about 1 s, therefore the first-order instrumentation should be used with a time constant of under a hundred milliseconds. The accuracy level of the surge control system should be 0.1% of the process measuring instruments. The changes around 1% in the input signal can be resolved by the recycle valves (final elements); therefore, the resolution of the process variables must be 0.1% of the standard range of operation. The resolution is corrupted by the over-ranged transmitter [6].

### 3.3 Flow-measuring devices

For surge prevention control, accurate flow measuring devices are easily available in the market; however, the system is slowed down by the transmitters. The response of the DP transmitter depends directly upon the strength of the signal as its range varies inversely to the response time. Hence, strong DP signal-generating devices are required. The signal-to-noise ratio is less in the devices having low-range signals. The response time is slow for the transmitters having low ranges of DP signal. The devices, like orifices, exhibiting the property of creating expansion and contraction to gas, can cause noise and turbulence. The flow measuring device is preferably installed on the compressor suction side [17].

### 3.4 Surge control valve

The controller response to sudden changes and slow changes for the surge condition has been discussed already. Similarly, the operation of the valve must be according to these two operations. The action should be of the order of a small valve or smooth action in the case of a slow change. But it must act fast just like a big valve in the case of a sudden change to overcome the surge in no time.

The properties of the valves have been categorized into three types.

1. Quick Action: The maximum capacity is obtained during the travel at the start.
2. Linear: The opening is linear to the capacity of the valve.
3. Equal Percentage: The maximum capacity is obtained at the end of the travel.

In the recycle valves, all of the above-mentioned types are utilized.

**Fig. 10:**
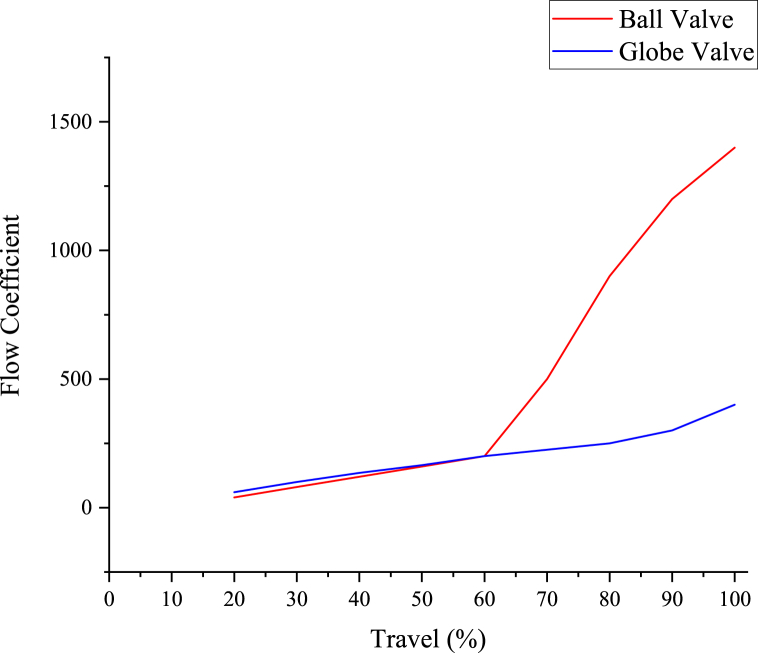
Comparison of ball and globe valves [6].

Noise-attenuating ball valves and equal percentage valves are preferred for the surge prevention methods, using only one control valve operating against the surge. At the near-to-close, these act as smaller valves but at the near-to-fully open, these act as bigger valves. The two different types of equal percentage valves are compared in Fig. 10. The globe valve is 0.5 times less expensive than the noise-attenuating ball valve for the required sized valve, but the latter gives the flow coefficient (Cv) three times more. Moreover, the reliability of the ball valve is higher, as the maintenance errors are negligible [5].

If the valve having equal percentage property is installed, the surge may be avoided for the shutdown case by providing enough capacity, also the firmness is maintained for the capacity below 50% for the solid control over the partial recycle action. For the compressor fitting, the resolution of the valve is greater for the equal percentage property than the linear property [13].

### 3.5 Valves arrangement

**Fig. 11:**
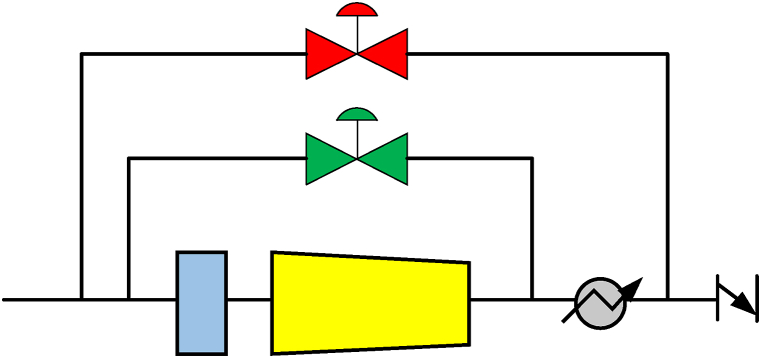
Arrangement of recycle valve for cold and hot [6].

On any side of the compressor, if the volumes are bigger, then the requirement of multiple valves is approached. The valve capacity may be reduced for the integrated method. The configuration of the recycle valves for hot and cold conditions is mentioned in Fig. 11. For quick action, the inner or the hot valve is on-off based while the outer or cold valve is the controlling type. The sizing of these valves is done individually. The shutdown valve capacity is also like the cooled valve if the cooled valve is a solenoid-type valve [5].

**Fig. 12:**
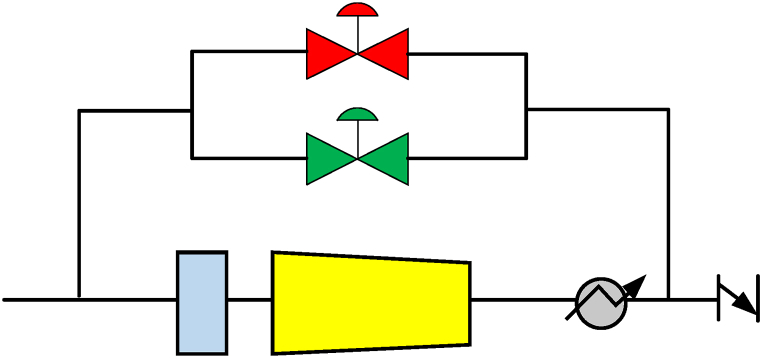
Arrangement of valves in parallel [6].

Another scenario can be made where an additional valve can be installed in parallel to the existing one as shown in Fig. 12. The redundancy factor is added to this type of configuration. The operation of these two valves is done in a cascade in the control. That implies the different setpoints being used by the valves like a surge margin of 9% or 10%. Usually, the 10% surge margin valve, which is considered a primary valve, is operated for the normal operating points. But contrary to this, if the movement is fast enough to beat the primary valve, then the secondary valve or the 9% surge margin valve will be operated. The redundancy is also provided in such a way that during the failure of the primary valve, the secondary valve is operated, hence acting as a primary valve in an emergency. This alteration can be done during the running of the compressor [6].

### 3.6 Actuation of valve

Rapid depressurization and modulating control are the two situations of operation for the surge prevention method in the shutdown, as discussed above. Therefore, to operate the above situations, a three-way solenoid valve is installed which can take the digital 24V signal to open for rapid depressurization or analog varying 4–20 mA standard signal for the modulating control.

The selection of the actuation system is the major difference between the normal valve and the surge control valve. Normally open or spring return is the recommended actuator for surge prevention. The method is reliable, simple, and best for the protection of the compressor in case of power failure. Actuators having a spring with a piston and a spring with a diaphragm, are used equally. The application of the diaphragm-type actuators is done on the globe valves. While the ball valves are preferred for the piston-type actuators. The rotary valves require powerful piston-type actuators as they need to drive the heavy mass ball using a larger force. Ball valves have weaker shafts and a weak connection to the ball therefore, not able to provide enough force for the operation of valves at a brisk pace, hence not suitable for surge prevention controls [17].

The opening action of the surge control valves must be very quick. For the same reason, the actuator must have wide air passages, strong springs, and near the end of travel, some shock absorbers as well to handle these speeds. This must be considered in surge control during the actuation of recycling valves.

### 3.7 Sizing of recycle valve and other components

The compressor operating conditions decide the sizing of the recycle valve. For the correct selection of the recycle valve for a compressor, the program of valve sizing can assist. The parameters of the compressor, including the process variables like speeds, pressures, heads, and temperatures, are fed into the tool. Similarly, the cooler details, relief valve, pipe pressures, operating speeds and temperatures, and other operating conditions are also fed. The data is then used by the tool to calculate the flow coefficient *C*_*vs*_ or valve capacities.

Usually, the valve capacity is merely constant for the surge limit of a compressor. The suppliers of the surge control valve can provide parameters: *C*_*v*_, *X*_*t,*_ and *C*_*g*_ values for the selection of the valve. Equal percentage property is used for a single surge control valve as mentioned already. After the selection of the valve, different opening conditions are operated by designing performance lines for a compressor map. During the surge, the travel of the equal percentage valve must be at the two-thirds level.

ISA standard method is used for the calculation of the flow rate. Where the standard flow is denoted by *Q*_*std*_, and the geometry factor of the piping is given by *F*_*p*_. Its value is taken as one and is not known on certain occasions. The pressure throughout the volume of the pipe is taken as persistent for the discharge pressure and the valve upstream.

(6) $$\frac{{dp}_{2}}{dt}=\frac{k.p_{2}}{V}\left[Q-Q_{V}\right]$$

(7) $$Q_{std}=1360.\;F_{p}\;.\;c_{v}\;.\;Y\;.\;{\left[\frac{p_{2}-p_{1}}{p_{2}}\;.\;\frac{1}{SG\;.T_{2}\;.\;Z_{2}}\right]}^{\frac{1}{2}}$$

(8) $$Q_{V}=Q_{std}\frac{\rho_{std}}{\rho \left(p_{2},T_{2},Z_{2}\right)}$$

Using the state equation of the Redlich-Kwong, the *Z*_*2*_ (Compressibility) is designed. It is clear from the above equations that the discharge pressure can only be greater if the valve is of greater capacity to pass through a bigger pressure, it doesn't depend upon the compressor's outcoming flow entirely. The drop in the pressure of a valve highly depends upon the size of the pipes i.e. fewer pressure drops in bigger volume pipes. The validation of the model is elaborated in Ref. [2].

The operating point of the compressor is involved in the calculation of the discharge pressure.

(9) $$\frac{p_{2}}{p_{1}}={\left[1+\frac{k-1}{k}\;.\;\frac{h\left(Q,N\right).SG}{287.ZT_{1}}\right]}^{\frac{k}{k-1}}\;\frac{h}{N^{2}}=\alpha {\left[\frac{Q}{N}\right]}^{2}+\beta \frac{Q}{N}+\gamma$$

In parallel to this, the flow and head relationship is obtained using a lookup table.

During ESD, the compressor behavior directly relies upon the two properties. The turbine, coupling, and compressor inertia (*J*) are one of two properties whose effect is evident in the ESD condition. The other parameter is the torque that directly involves the fluid. The torque is calculated as:

(10) $$T=-2\pi \;.\;J\;.\;\frac{dN}{dt}$$

The gas power can be calculated, using the torque and the inertia, with the alteration in the speed, as shown below:

(11) $$P=T\;.\;N\;.\;2\pi =-{\left(2\pi \right)}^{2}\;.\;J\;.\;N\;.\;\frac{dN}{dt}$$

The proportionality factor (*k*) remains unvaried for the case of speed and power regardless of the event occurrence of the rundown at any operating point. Similarly, the deceleration rate during the shutdown, which is calculated using k and *J*, doesn't depend upon the compressor operating point. The compressor head at a fixed speed is increased more when the surge margin is higher during the trip moment.

The recycle valve flow coefficient (*Cv*) is further divided into two parts considering the steady-state flow that is used in the normal operation and the flow in the case of the surge that is available at the time of the surge occurrence, i.e. *c*_*v,ss,*_ and *c*_*v,avail*_ respectively.

The *C*_*V, SS,*_ and the *C*_*V*_ of the recycle valve are known along with the first stream. Hence the available flow coefficient portion which can be used for the reduction of the backpressure can be calculated as below:

(12) $$c_{v,avail}=c_{v}-c_{v,ss}$$

The compressor is usually operated at a fixed temperature and most compressors have coolers to cool down the system. The coolers' thermal capacity is much bigger than the gas therefore the temperature variations during 1 s are near zero. The above equation result for the flow calculation is deducted from the discharge-occupied gas, resulting in piping volume and new pressure. This interpretation yields the maximum available actual volume of the piping under the given constraints where the surge is completely avoided during the ESD, which is also verified by the [2] using testing data. The expansion of this method is done for the volumes smaller on the suction side that exhibit more pressure rising rate on the suction side [6].

## 4 Fault-tolerant control systems

Industries having automated controls will face shutdown and unwanted operations, whenever a fault occurs in the field, and the resulting damage could lead to a huge loss in the cost of the equipment, machinery, and manpower. Process faults including sensor, actuator, and controller failures may lead to malfunctioning errors and may lead to unbearable and uncontrolled scenarios [72,73]. Similarly, the compressor ASC relies upon various sensors and an actuator. Any failure in the sensor will generate undesired results and the control action will disturb the system response causing severe damage to the machinery and human life due to the surge. Similarly, if the actuator fails to operate during the control action, the whole scenario is disturbed, and this failure will cause the same effect as if there is no ASC system installed in the system.

Fault tolerance is the capacity of a system to continue operating normally even if one or more of its components malfunction. A fault-tolerant control (FTC) is an automated control where the fault is accommodated using an advanced technique in which a system will continue to run despite the failure in the components and the system reliability will be enhanced. The overall performance and reliability are compromised when a fault occurs in any system. The systems in which the fault tolerance time is negligible are suitable for the FTC. FTC is implemented on the existing control system where the fault is detected and isolated and the process control is uninterrupted [74]. Fault tolerance should be considered in the sensitive elements of the system where the system's criticality is highly dependent upon it. Sensors and actuators of the system are the major elements that need to be addressed as these elements play a major role in the failure of the system by sending false information in the case of the faulty sensor and doing the wrong action in the case of the faulty actuator. Such control is handy in aircraft systems as in Ref. [75].

A fault-tolerant design (FTD) enables a system to continue running as intended, perhaps at a reduced level, rather than failing to operate completely, when a component fails. One of the cornerstones of a fault-tolerant control system (FTCS) is redundancy, which may be broken down into two types: hardware and analytical. The use of additional hardware to serve as a backup component and fulfill the same purpose is known as hardware redundancy. When using two redundant components, the main component controls all duties and switches to the backup component if there is an issue. A system's single point of failure, where a problem with one component might lead to the system failing altogether, is avoided when it has dual redundancy. However, hardware redundancy increases the system's price, mass, and footprint. Building a software model of the component is required for analytical redundancy in order to generate the sensor's virtual value in the event that the real hardware fails. Applications like those involving UAVs and airplanes may be able to reduce costs, weight, and physical dimensions with the use of analytical redundancy [76].

### 4.1 Types of fault-tolerant control systems

There are two main types of FTCS: analytical redundancy based and hardware redundancy based as discussed earlier. Their details are mentioned below.

#### 4.1.1 Analytical redundancy based FTCS

Two types of FTC are popularly known, namely passive fault-tolerant control system (PFTCS) and active fault-tolerant control system (AFTCS). The FTC details can be reviewed in Refs. [[77], [78], [79], [80], [81], [82]].

##### 4.1.1.1 Passive fault-tolerant control systems

**Fig. 13:**
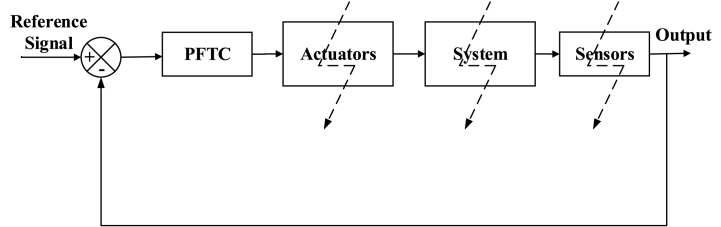
Passive Fault-Tolerant Control Schematic [75].

The PFTCS is a quick method where the controller is programmed in the design stage and is robust to other parameters and works irrespective of the faults. The PFTCS applications can be seen in Refs. [[83], [84], [85], [86], [87]]. A general schematic of the PFTCS is described in Fig. 13. The most common PFTCS recently is the sliding mode control due to its benefits of sturdiness to model uncertainties, system parameter variations, and external disturbances [[88], [89], [90], [91]].

##### 4.1.1.2 Active fault-tolerant control systems

**Fig. 14:**
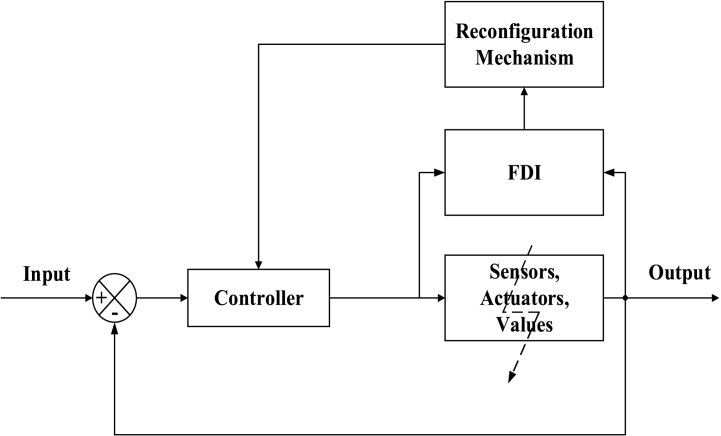
Active Fault-Tolerant Schematic [92].

AFTCS has a separate unit installed named fault detection and isolation (FDI) in which the fault is detected and isolated primarily. The configuration of the controller is redefined after the fault is treated by FDI [75]. AFTCS is a slow process, in comparison to PFTCS, due to its complicated structure and design. A general schematic of the AFTCS is shown in Fig. 14.

As the AFTCS has an issue with the delay in time and is slow due to more computations by the FDI as compared to the PFTCS, one can consider installing both AFTCS and PFTCS in parallel to work together to form a hybrid FTC. Such a system will be more efficient as compared to the AFTCS or PFTCS performing individually for a particular system. Hybrid FTC decreased the chance of failure with no time delay. In Ref. [92], an AFTCS is implemented for the air-fuel ratio control for the sensor and actuator faults using MATLAB software. Hybrid FTCS has been designed and implemented in Refs. [[93], [94], [95], [96], [97], [98], [99]]. In Ref. [100], a hybrid FTC is implemented for the air-fuel ratio of the engine for sensor and actuator faults. The FDI is implemented which is a part of AFTCS, while a robust controller is designed using Kalman Filter to make the controller robust against sensor and actuators’ noise. In Ref. [101], an effective FTC is used with the help of the linear matrix inequality (LMI) for the actuator faults in the flight control system. LMI has eliminated the need for FDI. In Ref. [102], a fault-tolerant wind turbine model is proposed in which different techniques have been presented for the fault detection and accommodation for the sensor, actuator and other system faults. In Ref. [103], an AFTCS is proposed for the sensor and actuator faults that is implemented on the wind turbine system. The compensated values for the faults are a bit degraded but the system prevents its complete failure. In Ref. [104], a fault tolerance has been proposed in the converters that is handy in the ac transmissions, therefore, providing a fault-tolerant system by implementing the redundant H-bridge building block for cascaded H-bridge multilevel converter.

In [105], FTC is implemented with the availability of online allocation using slide mode control where the position of the actuator is reallocated and the reconfiguration is not required for the controller. The review of sliding mode control from the perspective of FTC is elaborated in Ref. [106]. In Ref. [107], using neural networks, an adaptive design for tracking is implemented, with the help of the Lyapunov approach, which gives an estimate function for the fault, hence the efficiency of FTC is boosted using adaptive control design. Their work is done for the nonlinear system only. In Ref. [108], FTC is implemented by the sliding mode approach with observer-dependent control in which the input noise, sensor faults, and actuator faults are dealt with. Any fault in the sensor and actuator is tolerated simultaneously. In Ref. [109], the controller is designed for the attitude tracking of spacecraft in which the inertia calculations are not required which makes their model more efficient and quick, moreover, the actuator faults are removed using this technique. The application of AFTCS can be reviewed in Refs. [[110], [111], [112], [113], [114], [115]].

The control system functionality and the stability limitations can be proved using mathematical analysis of that control system. Hence analyzing the faults mathematically is necessary for both sensors and actuators due to their vulnerability to faults quite often. AFTCS is mathematically analyzed for a better understanding of the working of the controller to the readers. As the main function of the FTC is to cope with faults, therefore, a brief description of the faults and failures is explained for the sensors and actuators.

Mathematical modeling of observer design for sensor and actuator faults and modeling of faults can be seen in Refs. [[116], [117], [118]]. Faults in sensors are described below:

Bias: This is an offset value that is present in the sensor's output. Precisely, it is stated as Y=X+β and X and β are the true offset and sensor's offset values, respectively. The reason behind this can be corrosion of the sensor physically and improper calibration.

Drift: This is a mathematical linear and nonlinear deviation in the system.

Scaling: This error is of multiplicative type during the scaling procedure of the sensor, and it is described in the form of slope: Y(t)=α(t)X and α(t) is the time-varying scaling factor and ranges from 0≤α(t)≤∞.

Noise: This can be caused due to certain factors including random behaviors in hardware, wires, sensors, etc.

Hard Fault: The hard fault is fixed. Statistically, Y(t)=C and C represent a constant value here. For the complete failure of the sensor C=0 and the rest of the values represent the fixed value.

Generally, sensor faults are listed as additive and multiplicative types. The further mathematical explanation is described next:

Additive Fault:

(13) $$y^{f}=y+f_{y}$$

here $y^{f}$ represents the actual output fault value, y represents the output of the system, and $f_{y}$ describes the fault value of the sensor.

Multiplicative Fault:

(14) $$y^{f}=\left(1-\rho_{y}\right)y$$

here $\rho_{y}$ symbolizes fault gain, and ranges $0\leq \rho_{y}\leq 1$.

Combining the multiplicative and additive faults, it is obtained:

(15) $$y^{f}=\left(1-\rho_{y}\right)y\left(t\right)+f_{y}$$

Actuator fault types are as follows:

Lock failure: A complete failure, jamming, or locking of the actuator is a lock failure.

Float failure: In this fault, the actuator floats freely and varies its value by free motion.

Runaway or Handover: In this fault, a maximum or a minimum position of the actuator is attained.

Loss of Effectiveness: This is caused due to the material damage to the actuator causing it to stop.

Generally, the actuator faults can also be statistically represented by combining additive and multiplicative faults as mentioned:

Additive Fault:

(16) $$u^{f}=u+f_{u}$$

and $u^{f}$ represents the output of the actuator, u represents the input and $f_{u}$ denotes the fault value of the actuator respectively.

Multiplicative Fault:

(17) $$u^{f}=\left(1-\rho_{u}\right)u$$

where $\rho_{y}$ represents fault gain and $0\leq \rho_{y}\leq 1$.

Combining Additive and Multiplicative Fault:

(18) $$u^{f}=\left(1-\rho_{u}\right)u+f_{u}$$

A non-linear system is considered:

(19) $$\dot{x}\;\left(t\right)=Ax\;\left(t\right)+Bu\;\left(t\right)+g\left(x,u,t\right)$$

(20) y(t)=Cx(t)

$x\in R^{n}\;is\;state\;vector,u\in R^{p}\;is\;input\;vector,y\in R^{m}\;is\;output\;vector\text{.}$ A B and C are known and represent system matrices of proper dimensions. Where “n”, “p”, and “m” are the number of states, inputs, and outputs respectively.

g(x,u,t) represents a nonlinear function vector lying on $R^{n}$,

(21) z(t)=Cx(t)

z(t) is output fabricated variable.

Combining all the sensor faults:

(22) y(t)=z(t)+f(t)

(23) y(t)=(1−ρ(t))z(t)

(24) y(t)=z(t)−ρ(t)z(t)=z(t)+f(x,t)

(25) $$y_{i}\left(t\right)=z_{i}\left(t\right)+f_{i}\;\left(x,t\right)=C_{i}x\;\left(t\right)+f_{i}\;\left(x,t\right)$$

g(x,u,t) is supposed to be universally Lipschitz,

(26) $$\left\|g\left(x_{1},u,t\right)-g\left(x_{2},u,t\right)\right\|\leq \lambda \left\|x_{1}-x_{2}\right\|\forall u,t$$

λ represents a Lipschitz constant.

As per the defined value of the system, the observer provides a redundant value for the FDI. Therefore, it is the most important part of the FDI scheme. It is further explained mathematically below.

Observer design generally in mathematical form can be written as follows [119].

(27) $$\dot{x}=Ax+B\;u$$

(28) y=Cx

The observer equation is written mathematically as:

(29) $$\dot{\bar{x}}=A\bar{x}+B\;u+L\left(\bar{y}-y\right)$$

L denotes a state feedback gain matrix.

(30) $$\bar{y}=C\bar{x}$$

$\bar{x}$ denotes the estimated state value and $\bar{y}$ denotes the estimated output. $\bar{x}–x=e_{x}$ which is the error in the actual and estimated state of the system.

Subtracting (27) from (29), (28) from (30), it is obtained,

(31) $$\left(\dot{\bar{x}}-\dot{x}\right)=A\left(\bar{x}-x\right)+L\left(\bar{y}-y\right)$$

(32) $$\left(\bar{y}-y\right)=C\left(\bar{x}-x\right)$$

Rearranging (31) and (32), we get

(33) $$\left(\dot{\bar{x}}-\dot{x}\right)=\left(A+LC\right)\left(\bar{x}-x\right)$$

(34) $${\dot{e}}_{x}=\left(A+LC\right)e_{x}$$

Subtracting

(35) $$\left(\bar{y}-y\right)=Ce_{x}$$

The error vector $e_{x}$ declines to zero for a stable system and L can be calculated for the observer.

#### 4.1.2 Hardware redundancy-based FTCS

Real physical hardware redundancy is used in the hardware redundancy-based FTCS concept. Normal operation only requires one sensor for control purposes, but the terms double redundancy and triple redundancy, for instance, refer to the use of two or three sensors rather than one to measure the same quantity. Even if the primary hardware component fails, these backup components guarantee the system's reliability. To determine which channels are doing well and which ones are not, a voting system may be created. The actuators are also included in this idea of hardware redundancy. Since dual redundancy delays system failure until both components have failed, it improves the entire system's reliability. Even if a single component breaks one at a time, the system will still function. One of the high-reliability hardware designs in the hardware redundancy industry is the Triple Modular Redundancy (TMR) architecture. Voting among three contemporaneous channels that are working to provide results determines the final outcome. When a problem is detected by the voting mechanism in one of the channels, the other two channels are used to determine the outcome. Modified TMR (MTMR), a more sophisticated kind of TMR that can function even if two channels fail, was presented in Ref. [82].

### 4.2 Fault-tolerant control in anti-surge control

**Fig. 15:**
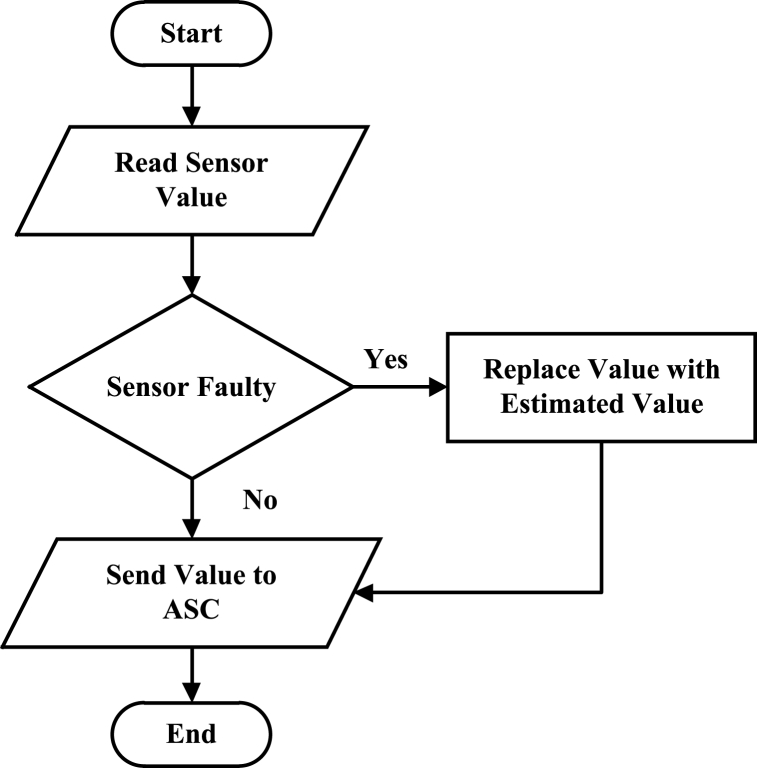
Fault-tolerant anti-surge control system.

The failure of the sensors and the actuator in the anti-surge system can occur for which the fault tolerance can be incorporated. A system is proposed where the AFTCS is integrated to compensate for the faulty sensors in ASC systems. When any sensor becomes faulty, its erratic value will be replaced by the virtual sensor, thereby, providing the analytical redundancy to increase the reliability of the system regardless of the faulty condition of the sensors [76]. The above scheme can be described in Fig. 15.

Several other applications can be implemented in the observer model of AFTCS in anti-surge control systems. For example, a support vector machine (SVM) and chaos sparrow search-based control is applied to surge prevention in blast furnace fans [120]. The proposed fault-tolerant system will detect the fault in a sensor and replace the faulty sensor value with the virtual values. The virtual values will be calculated with the help of SVM and chaos sparrow search. The analytical redundancy can be created based on the first principle modeling approach in which we mathematically model the whole system and develop mathematical observers as per AFTCS architecture as explained earlier. The latest trend is applying data-driven methods in which we develop models from the input and output data of the system. The main difference is the computational cost of the system as advanced data-driven methods are computationally heavy and require more sophisticated controllers. The unification of hardware redundancy with analytical redundancy will make the system highly reliable but costly at the same time. So a thorough cost and benefit analysis will have to be done before installing the unified FTC system.

### 4.3 Future research directions

From a future perspective, there is a clear research gap in terms of avoiding the chances of failure in the sensors of the ASC system. For this, fault tolerance is proposed to be combined with the existing system in which the faulty sensor will be traced by the FDI unit, and the faulty value will be replaced by the virtual value of the sensor. In this way, the system will not be affected by the failures and continue to operate at optimum conditions. This approach will increase the reliability of the system as virtual redundancy is added. For industries, where the compressors are the key units and the losses, are being measured in seconds, such failures will lead to severe damage unless these are procured by the proposed method of fault-tolerant ASC systems.

## 5 Conclusions

In this paper, a review of the ASC for the centrifugal compressor was presented. Several techniques were analyzed which addressed measuring the parameters like speed, flow, head, temperature, VFDs, surge lines, and flow speed ratio for surge detection and prevention. Some advanced techniques based on PLCs and fuzzy logic were also highlighted. A review of fault-tolerant control was also presented along with its types and mathematical formulation and future research directions. Fault tolerance for the ASC systems was proposed and it is concluded that for any compressor surge control system or ASC system besides the sizing, algorithms, and controlling parameters, some other aspects like the failure of the sensor and actuator, are to be considered as well to minimize the damage during the surge and maximize the efficiency of the compressor during any fault. All the above-mentioned parameters were also discussed, and this paper proposed a novel idea of applying fault tolerance in ASC systems.

## Funding

This research was supported by the Deanship of Scientific Research at 10.13039/501100005911Najran University through a grant (10.13039/501100005911NU/RG/SERC/12/29).

## Declaration of competing interest

The author(s) declare no conflict of interest in preparing this paper.
